# Implication of different domains of the *Leishmania major* metacaspase in cell death and autophagy

**DOI:** 10.1038/cddis.2015.288

**Published:** 2015-10-22

**Authors:** M Casanova, I J Gonzalez, C Sprissler, H Zalila, M Dacher, L Basmaciyan, G F Späth, N Azas, N Fasel

**Affiliations:** 1 Infections Parasitaires, Transmission et Thérapeutique, UMR-MD3, Aix-Marseille University, Marseille, France; 2 Department of Biochemistry, University of Lausanne, 155 Chemin des Boveresses, 1066 Epalinges, Switzerland; 3 Institut Pasteur and Centre National de la Recherche Scientifique URA 2581, Unité de Parasitologie Moléculaire et Signalisation, Paris, France

## Abstract

Metacaspases (MCAs) are cysteine peptidases expressed in plants, fungi and protozoa, with a caspase-like histidine–cysteine catalytic dyad, but differing from caspases, for example, in their substrate specificity. The role of MCAs is subject to debate: roles in cell cycle control, in cell death or even in cell survival have been suggested. In this study, using a *Leishmania major* MCA-deficient strain, we showed that *L. major* MCA (LmjMCA) not only had a role similar to caspases in cell death but also in autophagy and this through different domains. Upon cell death induction by miltefosine or H_2_O_2_, LmjMCA is processed, releasing the catalytic domain, which activated substrates via its catalytic dyad His/Cys and a proline-rich C-terminal domain. The C-terminal domain interacted with proteins, notably proteins involved in stress regulation, such as the MAP kinase LmaMPK7 or programmed cell death like the calpain-like cysteine peptidase. We also showed a new role of LmjMCA in autophagy, acting on or upstream of ATG8, involving *Lmjmca* gene overexpression and interaction of the C-terminal domain of LmjMCA with itself and other proteins. These results allowed us to propose two models, showing the role of LmjMCA in the cell death and also in the autophagy pathway, implicating different protein domains.

Apoptosis is, in most cases, associated with and depends on the activation of cys-dependent peptidases, named caspases.^1, 2^ Once activated, initiator caspases induce a proteolytic cascade via the activation of effector caspases that ultimately cleave numerous substrates, thereby causing the typical morphological features of apoptosis.^3, 4^ Despite their essential role in apoptosis, caspases are also involved in non-apoptotic events, including inflammation, cell proliferation, cell differentiation^5^ and the cell survival process autophagy, a major catabolic process in eukaryotic cells that allows cells to survive nutrient starvation due to engulfment of a portion of the cytoplasm by a specific membrane, delivery to lysosomes or vacuoles and digestion by hydrolytic enzymes.^6, 7, 8, 9, 10^ Plants, fungi and protozoa are devoid of caspases but express metacaspases (MCAs).^11^

MCAs are cysteine peptidases of the clan CD, family 14, with a caspase-like histidine–cysteine catalytic dyad.^12, 13^ However, besides their distant similarity to caspases,^14^ MCAs prefer arginine/lysine in the P1 position, whereas caspases prefer aspartic residues.^15, 16^ The role of MCAs in cell death is still enigmatic. For example, in the yeast *Saccharomyces cerevisiae*, YCA1 has a role in cell death,^17, 18^ whereas, although only partly dependent on its conserved catalytic cysteine, it also facilitates the removal of unfolded proteins, prolonging cellular life span.^19^ Similarly, some metacaspases have roles, outside of death, in stress acclimation pathways, as in *Aspergillus fumigatus*^20^ or in the unicellular planctonic organisms diatoms.^21, 22^ In *Arabidopsis thaliana*, AtMC1 is a positive regulator of cell death and a survival factor for aging plants,^23^ whereas AtMC2 negatively regulates cell death.^24^ *Trypanosoma brucei* TbMCA2, TbMCA3 and TbMCA5 and *Leishmania major* MCA are involved in cell cycle regulation.^25, 26^

*Leishmania* are parasitic protozoa responsible for the neglected tropical disease leishmaniasis, transmitted to humans by the bite of the sand fly. In the insect, parasites proliferate as free-living flagellated forms called procyclic promastigotes within the midgut before differentiating into virulent metacyclic promastigotes and migrating to the proboscis.^27, 28^ In the mammalian host, promastigotes are taken up by macrophages and transform into amastigotes. Under a variety of stress stimuli, apoptosis-like morphological and biochemical features have been described in *Leishmania*, among which are cell shrinkage, chromatin condensation, DNA fragmentation or mitochondrial depolarization.^29, 30, 31, 32, 33, 34, 35, 36, 37, 38^ Despite the evidence of morphological and biochemical markers of cell death in dying *Leishmania*, very little is known about the cell death pathway and the implicated executioner proteins. Indeed, essential proteins involved in mammalian apoptosis, death receptors, small pro- and anti-apoptotic molecules and caspases, are apparently not encoded in the genome of *Leishmania*^39^ and the role of *Leishmania* MCA in cell death is still controversial, certain authors suggesting a role as a negative regulator of intracellular amastigote proliferation, instead of having a caspase-like role in the execution of cell death.^40^

LmjMCA contains different domains: an N-terminal domain with a Mitochondrion Localization Signal (MLS),^41^ a caspase-like catalytic domain and a C-terminal proline-rich domain.^41^ On the basis of this domain structure, LmjMCA can be classified among the type I metacaspases,^16^ a subclass more generally defined in higher plants and characterized by the presence of an N-terminal prodomain and a short linker between the large and small subunits, as initiator caspases in metazoans.^11^ Upon induction of cell death by heat shock, H_2_O_2_ or drugs like miltefosine or curcumin, LmjMCA is processed and the catalytic domain is released,^41^ liberating the C-terminal domain. It was therefore interesting to investigate the functional roles of the different domains.

In this report, we studied the role of *L. major* MCA (LmjMCA), using an MCA-deficient strain and overexpressing independently the catalytic and the C-terminal domains. The results confirmed that MCA was not essential to *L. major* survival. In contrast, LmjMCA processing, releasing its catalytic and C-terminal domains, induced cell death in *L. major*, whereas the overexpression of *Lmjmca* gene triggered autophagy after interaction of the C-terminal domain with itself and with other proteins, acting on or upstream of the autophagic protein ATG8.

## Results

### Wild-type and MCA-deficient mutants have the same growth rate in normal conditions

**Figure 1:**
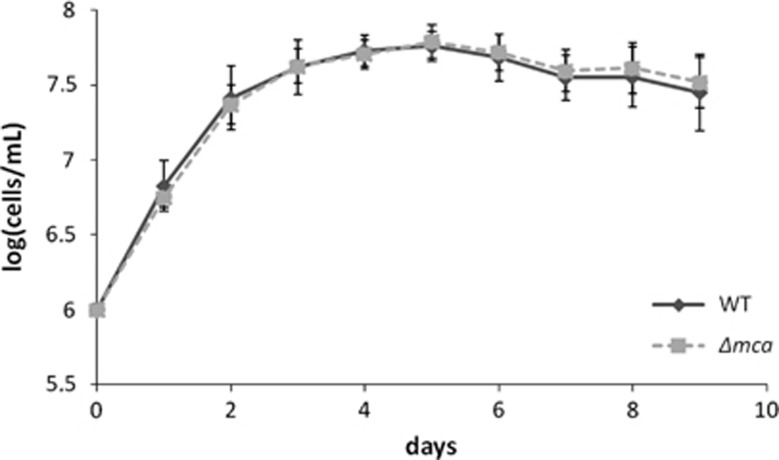
WT and LmjMCA-deficient strains have the same growth rate in normal conditions. Growth curves of WT (uninterrupted line with diamonds) and LmjMCA-deficient mutant cells (dotted line with squares; Δ*mca*) in normal conditions: means±S.D. from minimum five independent experiences. No growth difference could be detected between both strains in normal culture conditions

*L. major* MCA null mutants have been successfully generated thanks to the use of the Amaxa Nucleofector^40^ and the lack of the expression of the *Lmjmca* gene was confirmed by RT-qPCR (reverse transcription quantitative PCR, data not shown). We then monitored the growth of the promastigote form *in vitro*. No growth defect phenotype was observed as MCA-deficient cells grew at a rate comparable to the WT strain (Figure 1).

### *L. major* MCA is implicated in cell death

**Figure 2:**
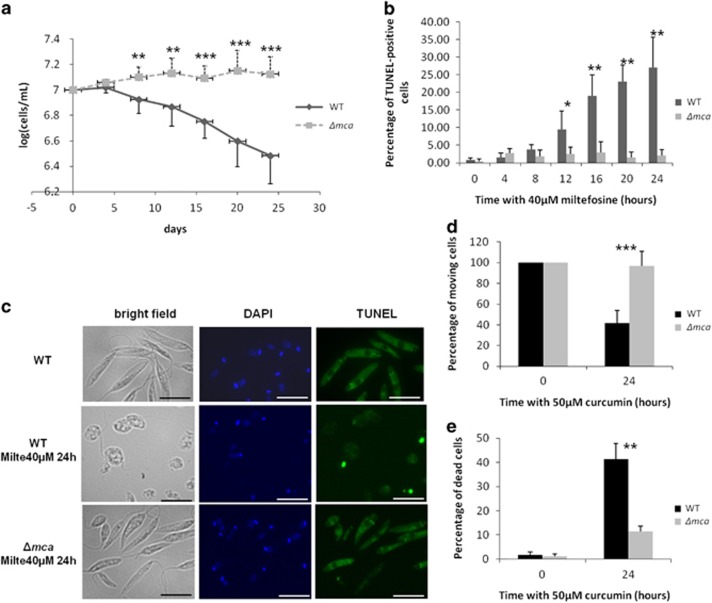
LmjMCA is involved in cell death. (**a**) Growth curves of WT (uninterrupted line with diamonds) and LmjMCA-deficient mutant cells (dotted line with squares; Δ*mca*) after the addition of 40 *μ*M of miltefosine: means±S.D. from minimum seven independent experiences. Significant growth differences could be observed between both the strains from 8 to 24 h. (**b**) Percentage of TUNEL-positive cells after the addition of 40 *μ*M of miltefosine for the WT (dark) and LmjMCA-deficient (gray) strains: means±S.D. from four independent experiences. A significant percentage of TUNEL-positive cells appeared in the WT strain from 12 to 24 h, whereas no TUNEL-positive cells could be detected when the LmjMCA-deficient strain was cultivated with 40 *μ*M of miltefosine. (**c**) Microscopical observation of WT cells in WT conditions (upper panels) and WT (middle panels) and LmjMCA-deficient (lower panels) strains after 24 h of cultivation with 40 *μ*M of miltefosine. From left to right, bright field, DAPI and TUNEL assay panels are presented. WT cells were clearly in apoptosis as shown by their rounded shape and their TUNEL staining, whereas the LmjMCA-deficient cells showed no different phenotype in comparison with the WT conditions and no TUNEL staining (scale bar, 10 *μ*m). (**d**) Percentage of moving cells after treatment of WT and LmjMCA-deficient cells with 50 *μ*M of curcumin for 24 h. We could observe a significant difference between the two strains, the curcumin having no effect on LmjMCA-deficient cells: means±S.D. from minimum five independent experiences. (**e**) Percentage of dead cells (TUNEL-positive cells and cells with no nucleus) after treatment of WT and LmjMCA-deficient cells with 50 *μ*M of curcumin for 24 h. Curcumin induced significantly less apoptosis in the LmjMCA-deficient than the WT strain: means±S.D. from three independent experiences. Unpaired *t*-test, *P<0.05, **P<0.01 and ***P<0.001

To induce cell death, we cultivated cells with 40 *μ*M of miltefosine over a 24-h period. Contrary to WT, MCA-deficient cells did not die in these conditions, as shown by significant growth differences between both strains starting 8 h after drug addition (Figure 2a). Cell death could also be correlated with the presence of positive cells in a TUNEL assay, a method allowing to detect DNA fragmentation.^42^ After incubation with 40 *μ*M of miltefosine, the percentage of TUNEL-positive cells increased from 0 to about 27% in the WT strain (Figure 2b) but stayed under 3% in MCA-deficient cells during the 24 h of treatment. Cell death was also visualized by fluorescence microscopy, where WT cells had a rounded shape when exposed to miltefosine as reported in Foucher *et al.*,^43^ whereas LmjMCA-deficient cells maintained an elongated shape (Figure 2c). To ensure that the different behavior of WT and LmjMCA-deficient strains was not owing to resistance of the second strain to miltefosine, we added curcumin, another anti-*Leishmania* drug.^44^ This drug induced significant growth defects in the WT strain, accompanied by a significant increase in the percentage of dead cells, but not in the LmjMCA-deficient strain (Figures 2d and e). These results indicated that LmjMCA had an important role in programmed cell death (PCD), induced by miltefosine as well as by curcumin.

**Figure 3:**
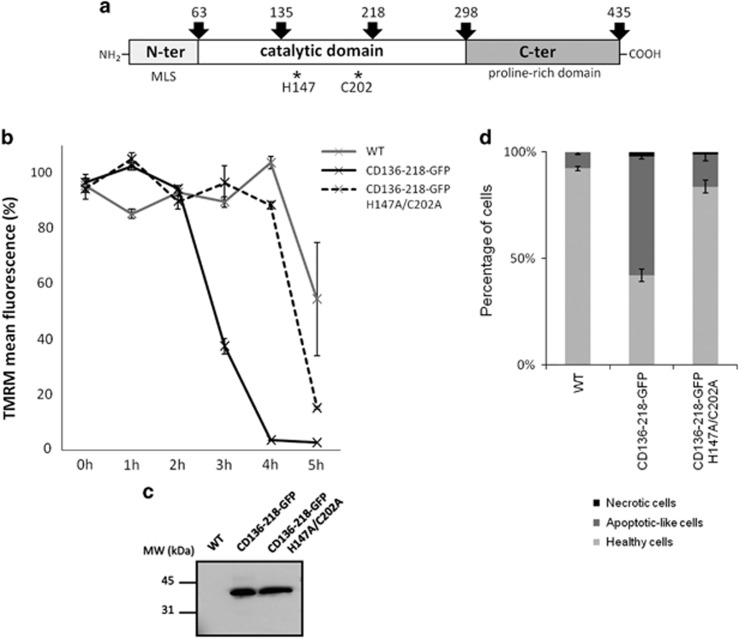
LmjMCA catalytic domain induces cell death. (**a**) Schematic representation of the *L. major* metacaspase. LmjMCA contains an N-terminal domain (aa 1 to 63) containing a predicted Mitochondrion Localization Signal (MLS), a caspase-like catalytic domain (aa 64 to 298) with the catalytic dyad (His147 and Cys202) and a C-terminal proline-rich domain (aa 299 to 435). (**b**) TMRM mean fluorescence representing the mitochondrial membrane potential of WT, CD136-218-GFP and CD136-218-GFP H147A/C202A parasites exposed to 0.5 mM of H_2_O_2_ (means±S.D. from three independent experiences). We could observe an important loss of mitochondrial membrane potential after 2 h for the CD136-218-GFP expressing cells, and not for the WT strain or for the strain expressing the inactive form of LmjMCA-CD. (**c**) Western blot expression analysis of GFP fusion proteins from WT parasites, parasites expressing CD136-218-GFP and parasites expressing CD136-218-GFP H147A/C202A (36 kDa), using an anti-GFP antibody. (**d**) Mitochondrial membrane potential and cell integrity analysis after a 3 h H_2_O_2_ treatment of parasites expressing WT LmjMCA, CD136-218-GFP and CD136-218-GFP H147A/C202A. Percentages of healthy (TMRM-positive, live/dead-negative), apoptotic-like (TMRM-negative, live/dead-negative) and necrotic cells (TMRM-negative, live/dead-positive) are represented by stacked columns (means±S.D. from three independent experiences)

LmjMCA contains an N-terminal domain (amino acids (aa) 1 to 63) with MLS, a caspase-like catalytic domain (aa 64 to 298) with the catalytic dyad (His147 and Cys202) involved in cell death^41^ and a C-terminal proline-rich domain (aa 299 to 435) (Figure 3a).^41^ We tried to define the shortest active form that was still able to induce cell death when overexpressed in the parasite and therefore to exclude any other domain. Auto-processing of LmjMCA precursor polypeptide at arginine residues releases different forms of CD-LmjMCA with molecular sizes ranging between 10 and 35 kDa.^41^ In view of the arginine sites in the catalytic domain, processing events at arginine 136 and 218 would generate a fragment of 9.1 kDa. Hence, we transfected parasites with a GFP-tagged polypeptide corresponding to aa 136 to 218 (CD136-218-GFP). Parasites overexpressing CD136-218 were exposed to 0.5 mM H_2_O_2_ to trigger cell death,^29^ and mitochondrial membrane potential was measured over time using the TMRM sensor.^42^ Mitochondrial depolarization was observed in cells overexpressing CD136-218–GFP after 2 h of H_2_O_2_ treatment in comparison with WT parasites, where an effect was observed only after 4 h (Figure 3b). This phenotype was reverted when the catalytic residues were changed to alanine (Figure 3b). The effect could not be imputed to different levels of expression as both CD136-218-GFP and its mutated version were expressed at similar levels (Figure 3c), therefore demonstrating a direct enzymatic activity-dependent effect of the short form of CD-LmjMCA in cell death induction. CD136-218-GFP clearly showed no significant increase in necrotic cells in comparison with the five-fold increase in apoptotic-like cells (Figure 3d).

We also monitored the expression of the *Lmjmca* gene in dying cells by RT-qPCR, showing that when cells were cultivated with 40 *μ*M of miltefosine during 24 h, no significant overexpression of the *Lmjmca* gene could be observed (Supplementary Figure S1).

### *L. major* MCA is implicated in autophagy

**Figure 4:**
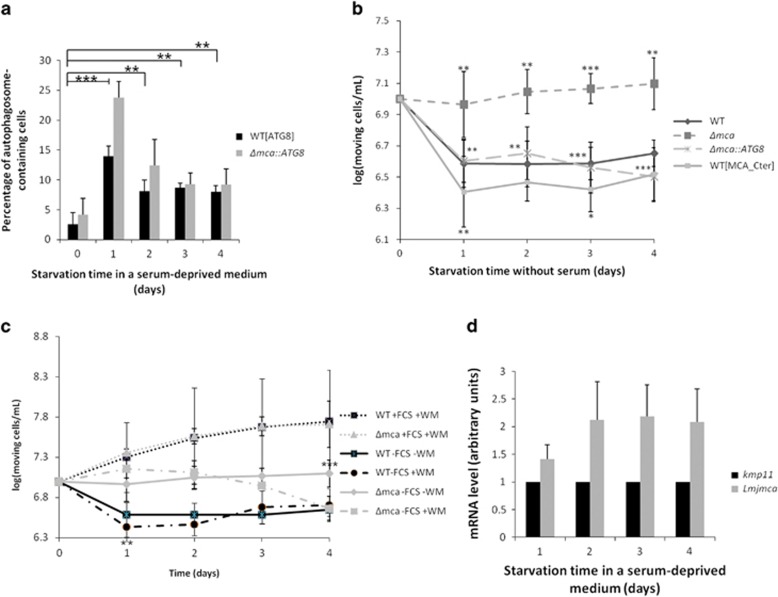
LmjMCA is involved in autophagy. (**a**) Percentage of autophagosome-containing (cells with a punctuated ATG8 staining) WT (dark) and LmjMCA-deficient (gray; Δ*mca*) cells cultivated in a serum-deprived medium: means±S.D. from four independent experiences. WT cells significantly entered autophagy when cultivated in a serum-deprived medium (***P<001, **P<0.01, compared with *t*0), whereas no significant difference could be detected between the percentage of WT and Δ*mca* autophagosome-containing cells. (**b**) Growth curves of WT (uninterrupted black line with diamonds), LmjMCA-deficient mutant cells (Δ*mca*; dotted line with squares), Δ*mca* cells expressing ATG8-GFP (Δ*mca::ATG8*; dotted line with crosses) and cells overexpressing the C-terminal domain of LmjMCA (WT[MCA_Cter] uninterrupted gray line with squares) when cultivated in a serum-deprived medium: means±S.D. from minimum seven independent experiences for WT, Δ*mca* and WT[MCA_Cter] and four independent experiences for Δ*mca::ATG8*. Significant growth differences could be observed for Δ*mca* compared with the WT strain, for WT[MCA_Cter] compared with the WT strain at days 1 and 3, and for Δ*mca::ATG8* compared with the Δ*mca* strain. (**c**) Growth curves of WT and LmjMCA-deficient strains in normal conditions with 10 *μ*M wortmannin (WT+FCS+WM, dotted black line with squares and Δ*mca*+FCS+WM, dotted gray line with triangles, respectively), of WT strains cultivated in a serum-deprived medium without (WT−FCS−WM, uninterrupted black line with squares) or with 10 *μ*M wortmannin (WT−FCS+WM, dotted black line with circles) and of LmjMCA-deficient cells cultivated in a serum-deprived medium without (Δ*mca*−FCS−WM, uninterrupted gray line with diamonds) or with 10 *μ*M wortmannin (Δ*mca*−FCS+WM, dotted gray line with squares): means±S.D. from minimum three independent experiences. We can note a significant effect of wortmannin at day 1 for WT cells and at day 4 for Δ*mca*, when cells are cultivated in a serum-deprived medium. (**d**) RT-qPCR quantification of *kmp11* (Kinetoplastid Membrane Protein, used as a control) and *Lmjmca* mRNA expression, after culture of WT cells in a serum-deprived medium (means±S.D. from minimum three independent experiences). We could note a significant overexpression of *Lmjmca* at day 3 and 4. Unpaired *t*-test, *P<0.05, **P<0.01 and ***P<0.001

To gain further insight into the functional role of LmjMCA, we investigated its role in the cell survival process autophagy, this process having been related to cell death (reviewed in Mariño *et al.*^45^). When WT cells were cultivated in a serum-deprived medium, they entered autophagy as demonstrated by the significant increase in the percentage of cells with puncta of the autophagosome marker ATG8-GFP^46^ (Figure 4a). We could note that autophagy was correlated with a significant decrease in the concentration of cells counted with a Thoma cell counting chamber (Figure 4b). Yet, no cell death occurred as demonstrated by the absence of TUNEL-positive cells (Supplementary Figure S2). Instead, the concentration decrease was owing to a fast loss of mobility (data not shown), whereas only moving cells were counted with the Thoma chamber.

In these conditions of autophagy, we could observe significant differences between concentration of WT and LmjMCA-deficient moving cells: the number of WT moving cells was decreasing before being stabilized, whereas the number of LmjMCA-deficient mutant cells that were moving remained the same (Figure 4b). This different behavior in autophagic conditions suggested the involvement of LmjMCA in autophagy. To confirm the involvement of LmjMCA in autophagy, we added wortmannin, an autophagy inhibitor.^47^ We observed that this inhibitor had no effect on cells cultivated in normal conditions, whereas it induced a significant decrease in concentration at day 1 when WT cells were cultivated in a serum-deprived medium (Figure 4c). This concentration decrease was owing to necrosis, characterized by the appearance of fragmented nuclei (data not shown). This indicated that autophagy occurred, notably at day 1, in serum-deprived conditions, whereas there was no autophagy in normal conditions. On the contrary, no necrosis was induced from day 1 to day 3 when LmjMCA-deficient cells were cultivated in a serum-deprived medium (Figure 4c), indicating the absence of autophagy in these mutants in the absence of serum. We could note that autophagy occurred in the LmjMCA-deficient line at day 4 since wortmannin induced cell death (Figure 4c). Growth differences in autophagic conditions and the absence of effect of the autophagy inhibitor wortmannin on LmjMCA-deficient cells cultivated in autophagic conditions indicated that LmjMCA was implicated in autophagy.

When we transfected the LmjMCA-deficient cells with ATG8-GFP, no differences could be observed between the percentage of ATG8-GFP puncta of WT and LmjMCA-deficient cells (Figure 4a). Furthermore, the LmjMCA-deficient strain expressing ATG8-GFP had the same growth curve as WT cells expressing the autophagosome marker (Figure 4b). This indicated that autophagy occurred in the deficient strain overexpressing ATG8, but not in the deficient cells not overexpressing it. As a consequence, as ATG8 could complement the lack of autophagy in LmjMCA-deficient cells, LmjMCA acted either on or upstream of ATG8, like the metacaspase of the Norway spruce embryo suspensor.^48^

Moreover, when cells were cultivated in a serum-deprived medium, a significant difference could be observed between the concentration of WT moving cells and cells overexpressing the LmjMCA C-terminal domain that were moving, notably at day 1, evaluated with a Thoma counting chamber (Figure 4b). The C-terminal domain of LmjMCA was thus responsible for the role of metacaspase in autophagy.

Furthermore, we demonstrated by RT-qPCR that the *Lmjmca* gene is about two times more expressed at days 2, 3 and 4 when cells were grown in a serum-deprived medium (Figure 4d). As a consequence, autophagy could be correlated to the overexpression of the *Lmjmca* gene. Unfortunately, we could not confirm the increased expression of LmjMCA at the protein level owing to lack of good quality antibody.

**Figure 5:**
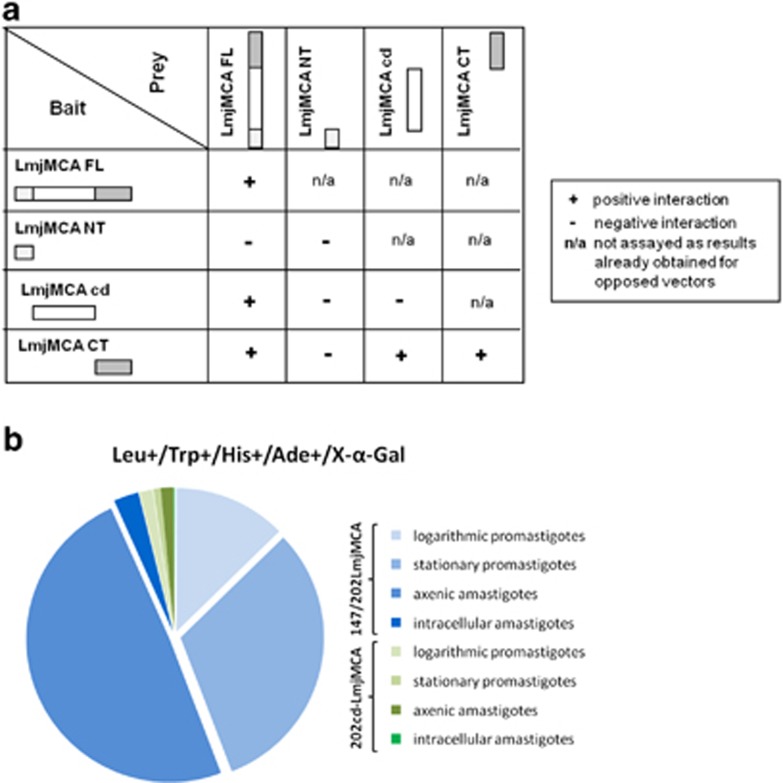
Yeast two-hybrid assay. (**a**) Self-interaction of LmjMCA via its C-terminal domain. Interactions of the LmjMCA full-length form (FL), the N-terminal domain (NT), the catalytic domain (cd) and the C-terminal domain (CT) have been tested against each other by cloning them into the bait and/or the prey vector for yeast two-hybrid assay. The positive interactions (+) seemed mainly owing to the C-terminal domain of LmjMCA. (**b**) Number of positive colonies growing in a medium without leucine, tryptophan, histidine and adenine and with X-*α*-Gal, co-transfected with different GAL4-AD protein libraries (logarithmic promastigotes, stationary promastigotes, axenic amastigotes and intracellular amastigotes) and either the inactive full-length LmjMCA (147/202LmjMCA, in blue) or the inactive catalytic domain of LmjMCA (202 cd-LmjMCA, in green). A greater number of positive colonies was obtained with the full-length than with the catalytic domain

A yeast two-hybrid assay was also performed. For this, yeast cells were co-transfected with the full-length LmjMCA or three different regions of LmjMCA fused either to a GAL4-activating domain or to a GAL4 DNA-binding domain. We analyzed the N-terminal region, the region comprising the catalytic domain and the C-terminal region. We used LmjMCA peptides mutated in the catalytic dyad instead of active forms to prevent LmjMCA auto-processing and the potential cleavage of the interacting protein.^41^ Selection of clones was obtained when full-length forms of LmjMCA were co-expressed, showing self-interaction of LmjMCA, mainly owing to the C-terminal domain as clones could be selected when the C-terminal domains were co-expressed or expressed with the catalytic domain (Figure 5a). Interestingly, when the positive self-interactions of LmjMCA were put into selective liquid medium, cells flocculated. Yeast flocculation being correlated to a response to conditions of nitrogen stress, it confirmed the involvement of the C-terminal domain of LmjMCA in autophagy.

### LmjMCA C-terminal domain interacts with proteins involved in stress regulation, PCD and vesicle transport

A yeast two-hybrid screening was used to identify *L. major* proteins that could interact with LmjMCA during different life cycle stages of the parasite, using cDNA libraries from logarithmic and stationary phase promastigotes and axenic or intracellular amastigotes. These yeast cells were simultaneously co-transfected with the inactive complete sequence or catalytic domain of LmjMCA. Most part of the 851 positive colonies was obtained with the full-length protein and very few with the catalytic domain (Figure 5b).

From the positive colonies, 86 proteins were identified, among which hypothetical proteins (Table 1) and proteins with function inferred from homology, experimental characterization or previous publication (Table 2 and Supplementary Table S1). Table 3 summarized the localization of these proteins and classified them in five groups: Ser/Thr protein kinases, proteases, proteins involved in vesicle transport, proteins involved in metabolism and nucleotide-binding proteins.

Of note, two proteins involved in stress response regulation or PCD were identified: the mitogen-activated protein kinase 7 (LmaMPK7; LmjF.13.1640) and a calpain-like cysteine peptidase (CALP; LmjF.27.0500). Remarkably, LmaMPK7 was identified in libraries from logarithmic and stationary phase promastigotes and CALP in libraries from stationary phase promastigotes and axenic amastigotes (Table 2 and Supplementary Table S1). In these two proteins, the aa sequences identified as interacting with LmjMCA corresponded to their catalytic domains (Table 2). Four proteins putatively involved in vesicle transport were identified as interacting with LmjMCA: the Rab1 small GTP-binding protein, which interacted with the inactive catalytic domain (Table 2), the dynein heavy chain, the ADP-ribosylation factor (Arf) GTPase activating protein (GAP) and the transport sec-23-like protein, which interacted with the full-length LmjMCA. Interestingly, the interactions of LmjMCA with the Arf GAP and with the transport protein sec-23-like gave flocculation of the yeast cells when put into a selective liquid medium, indicating that these proteins interacted only weakly or transiently with LmjMCA, not preventing self-interaction of the LmjMCA bait.

**Figure 6:**
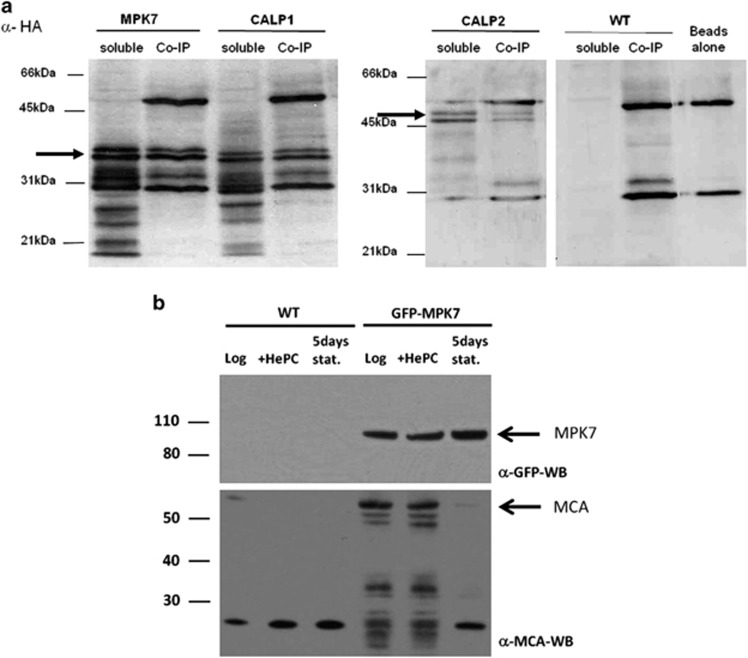
Confirmation of the interaction of MCA with LmaMPK7 and CALP. (**a**) Co-immunoprecipitation, confirming the interaction of LmaMPK7 and CALP with LmjMCA. Western blot of lysates (soluble) and immunoprecipitated beads (Co-IP), revealed with an anti-HA antibody, after cell lysis and incubation with protein-sepharose beads. The two prominent bands at around 50 and 30 kDa represent the heavy and light chain of the myc-antibody, respectively that has been coupled to the sepharose beads (beads alone). Out of five tested proteins, only two could be co-immunoprecipitated at a size of about 37 and 47 kDa (black arrows), namely the interacting sequence of LmaMPK7 and a first sequence of CALP (CALP1) at 37 kDa and a second sequence of CALP (CALP2) at 47 kDa. An unspecific band appears at around 32 kDa (WT). Two bands appeared for all of the co-immunoprecipitated proteins on which shrimp alkaline phosphatase treatment or incubation of the membrane with an ubiquitin antibody had no effect. The probable cleavage products obtained for the soluble fractions of LmaMPK7 and CALP1 could be reduced by adding a protease inhibitor cocktail, leupeptin, that inhibits LmjMCA^64^ and pepstatin as shown for CALP2, although much less proteins were present. Two bands were nevertheless appearing for the co-immunoprecipitated proteins. (**b**) Pull-down assay, confirming the interaction of LmaMPK7 with LmjMCA. Total proteins were extracted from non-transfected *L. major* WT cells and WT parasites expressing GFP-tagged *L. major* MPK7 (GFP-MPK7) obtained from logarithmic cultures that were untreated (log) or treated for 4 h with 40 *μ*M of miltefosine (+HePC), or from day 5 stationary phase (stat). GFP-MPK7 was purified, separated by SDS-PAGE and analyzed by immunoblotting with anti-GFP (*α*-GFP-WB) and anti-MCA antibodies (*α*-MCA-WB). Molecular mass standards are indicated in kilodaltons (kDa) (GFP-MPK7: 94 kDa, MCA: 54 kDa). The <30 kDa band represents a nonspecific signal as it is revealed in the control pull-down using anti-GFP antibody in combination with lysates from untransfected WT parasites

Co-immunoprecipitation experiments confirmed the interaction of LmjMCA with LmaMPK7 and CALP (Figure 6a) but not with Sec23-like protein, Arf GAP and Rab1 small GTP-binding protein. Further, the *E*-value for Rab1 being quite high (3e−11; Supplementary Table S1), a criterion which can be used to test the quality of the prey sequence,^49^ it is quite possible that the Rab1-LmjMCA interaction is a false positive, simply owing to a small Rab1 interacting sequence potentially homologous to other protein domains.

We confirmed the interaction of LmjMCA with LmaMPK7 by a pull-down assay (Figure 6b). Total proteins extracted from WT cells and GFP-tagged MPK7 transfected parasites, in normal culture conditions, after induction of cell death with miltefosine or induction of autophagy, were separated by SDS-PAGE after isolation of GFP-MPK7 confirmed with an anti-GFP antibody. LmjMCA could be isolated with GFP-MPK7 from cells cultivated in normal and apoptotic conditions, but not in autophagic conditions (Figure 6b). We did not realize pull-down assays on the other proteins identified by the yeast two-hybrid assay.

## Discussion

### LmjMCA, a role similar as the one of caspases in cell death

We demonstrated that LmjMCA induced cell death, involving both the catalytic and C-terminal domains, under different apoptotic stimuli (miltefosine, curcumin and H_2_O_2_). Indeed, we showed that the enzymatic activity of the LmjMCA catalytic domain ranging from aa 136 to 218 was essential for the induction of cell death. Furthermore, LmjMCA interacted with proteins involved in stress response regulation and cell death like LmaMPK7 and CALP. This interaction occurred with inactivated forms of LmjMCA and was mainly related to its C-terminal domain, which is a proline-rich C-terminal domain containing a WW binding domain motif, that mediates protein–protein interactions.^26, 50, 51^

Mitogen-activated protein kinases (MAPKs) are serine threonine protein kinases involved in cell growth, differentiation, gene expression, mitosis, cell motility, metabolism, cell survival and apoptosis.^52, 53, 54^ MAPKs phosphorylate substrates with the general consensus sequence P-X-S/T-P^54^ and LmjMCA has the PQSP motif (aa 369 to 372) in its C-terminal domain, suggesting that the phosphorylation by LmaMPK7 of the WW binding domain motif could regulate the interaction of LmjMCA with other proteins.

Calpains (calcium-activated papain-like proteases) are widely expressed cysteine proteases implicated in a broad range of cellular functions including proliferation, cell migration and apoptosis.^55^ In *Leishmania* species, calpain activity has been associated with PCD.^31, 56^ However, the calpain catalytic triad CHN is not conserved in CALP here identified, implying no cleavage of LmjMCA by CALP.

Last, we showed that an apoptotic stimulus did not induce any significant overexpression of the *Lmjmca* gene. We can thus formulate the hypothesis that LmjMCA involvement in cell death was related to its processing, confirming the observed processing of LmjMCA when cells were cultivated in cell death-inducing conditions.^41^

**Figure 7:**
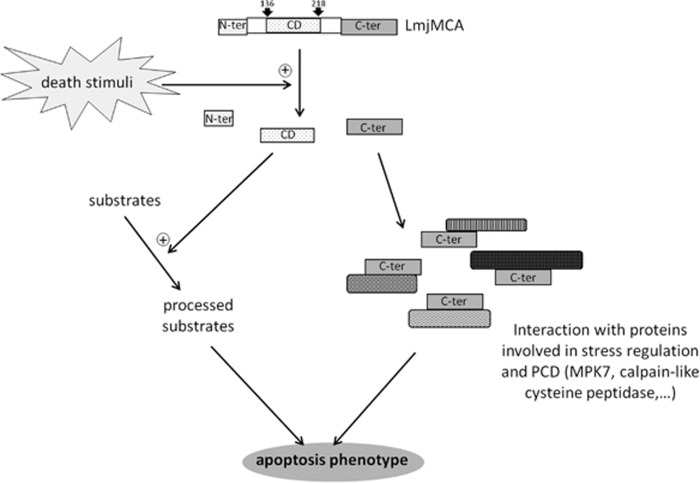
Model: role of LmjMCA in cell death. Apoptosis stimuli would induce LmjMCA processing, releasing the catalytic domain, notably the catalytic domain ranging from aa 136 to 218. The catalytic domain, via its catalytic dyad His/Cys, would enzymatically activate substrates through their processing, and the C-terminal domain would interact with proteins involved in stress regulation or programmed cell death like LmaMPK7 and the calpain-like cysteine peptidase, independently of any enzymatic activity. These two pathways would trigger the apoptosis phenotype

Figure 7 summarizes these results. In this model, cell death stimuli could induce LmjMCA processing, releasing the catalytic domain, notably ranging from aa 136 to 218, and the C-terminal domain. Then, two independent pathways could be induced: (1) the catalytic domain, via its dyad His/Cys, could enzymatically cleave substrates and trigger the apoptosis phenotype; (2) the C-terminal domain could interact with proteins involved in stress regulation or PCD like LmaMPK7 and CALP, triggering the apoptosis phenotype. This model proposes a role of LmjMCA similar to the one of caspases in cell death.^3, 4^ This confirmation of PCD in ancestral eukaryotes, as the identification in ancient lineages of marine phytoplankton (reviewed in ref. 57), places the origins of PCD far earlier than the rise of metazoans.

### A novel role of LmjMCA as autophagic trigger

We also showed that the C-terminal domain of LmjMCA induced autophagy, acting on or upstream of ATG8, and that autophagy was related to *Lmjmca* gene overexpression. We demonstrated that LmjMCA, mainly by its C-terminal domain, interacted with itself and with other proteins, among which, as suggested by the yeast two-hybrid assay, proteins possibly involved in vesicle transport: Rab1 small GTP-binding protein, dynein heavy chain, Arf GAP and transport protein sec-23-like. The interaction of the Arf GAP and of the transport protein sec-23-like with LmjMCA was weak and transient, not preventing LmjMCA self-interaction. The interaction of LmjMCA with these different partners suggests a possible role of this metacaspase in endoplasmic reticulum to Golgi transport in *L. major* parasites, possibly allowing autophagy via transport of autophagic proteins. This aspect will require additional investigation.

Interestingly, when the positive self-interactions of LmjMCA were put into selective liquid medium, cells flocculated. Flocculation is defined as asexual, reversible and Ca^2+^-dependent aggregation of yeast cells.^58^ It is mediated by specific cell surface lectins, able to bind directly to mannose residues of mannan molecules on adjacent cells.^59^ Flocculation of yeast cells is related to response to conditions of nitrogen stress.^60, 61^ As a consequence, the flocculation when co-expressing the C-terminal domains of LmjMCA confirmed the involvement of the C-terminal domain of LmjMCA in autophagy.

Of note, LmjMCA-deficient cells did not enter autophagy at day 1 but entered rather at day 4 (Figure 4c). We can hypothesize that few nutrients remained from the medium at day 1, whereas they must have been eliminated at day 4 owing to cell consumption. In this case, LmjMCA would induce autophagy when nutrient concentration would decrease, whereas autophagy would directly be induced without the involvement of LmjMCA when no more nutrients would be available.

**Figure 8:**
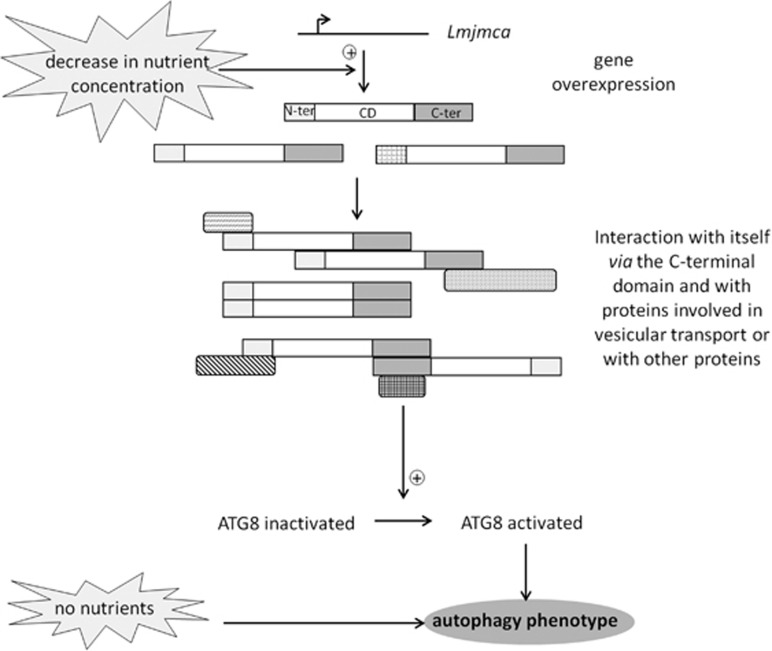
Model: role of LmjMCA in autophagy. A decrease in nutrient concentration would induce *Lmjmca* overexpression. The overexpressed LmjMCA proteins would interact, via their C-terminal domain, with themselves and with proteins involved in vesicle transport, inducing ATG8 activation and thus the autophagy phenotype. The absence of nutrients would induce autophagy directly, without involving LmjMCA

We propose the model presented in Figure 8 to link LmjMCA and autophagy in which a decrease in nutrient concentration would induce *Lmjmca* overexpression. Overexpressed LmjMCA proteins would self-interact, via their C-terminal domain and interact with other proteins, perhaps involved in vesicle transport, inducing autophagy through ATG8 activation. The absence of nutrients would also induce autophagy, without involving LmjMCA.

The results presented here highlighted the antagonistic roles of LmjMCA in PCD and in the cell survival process autophagy, as the metacaspases of *A. thaliana*^23^ and diatoms (reviewed in ref. 57). This would show a gathering of functions in one protein in ancestral unicellular organisms. However, there is ambiguity in autophagy/PCD relationships, brought about by a plethora of mechanistic intersections between the two processes, reviewed in Mariño *et al.*^45^ As a consequence, the two models here presented involving LmjMCA could constitute a unique pathway triggering both autophagy and cell death. However, this hypothesis needs further experimental evidence to be confirmed.

## Conclusion

In conclusion, we here confirmed the involvement of LmjMCA in cell death, either by the release of its catalytic domain or by interaction of the C-terminal domain with partners involved in stress regulation or cell death. We also identified a new role of LmjMCA in autophagy, in relation with gene overexpression and interaction of LmjMCA, mainly owing to its C-terminal domain, with itself and other proteins. These results open new perspectives on the role of MCA. The identification of the enzymatic substrates of LmjMCA and of the triggering stimuli would clarify the metabolic pathways involving LmjMCA and leading to cell death and/or autophagy.

## Materials and Methods

### Parasites

*L. major* wild-type parasites MRHO/IR/75 promastigotes were grown in Schneider's *Drosophila* medium supplemented with 100 U/ml penicillin, 100 *μ*g/ml streptomycin and 20% heat-inactivated fetal calf serum (FCS) (Gibco, Life Technologies, Saint-Aubin, France) at 26 °C. LmjMCA-deficient cells were a kind gift from Jeremy Mottram (Institute of Infection, Immunity and Inflammation, University of Glasgow) and were grown in the same medium with 30 *μ*g/ml hygromycin B (Invitrogen, Saint-Aubin, France).

### Molecular constructs

The DNA sequence encoding the LmjMCA sequence from aa 136 to 218 of LmjMCA was amplified using the primer pairs 136Fwd_NdeI (5′-GCGATAACATATGCCCGGTGATGTGCTTTTTTTC-3′)/218Rev_BglII (5′-CGCAGATCTCGTGGCCACGTAGCTGAAGG-3′) and LmjMCA-Flag construct^41^ as template, then inserted into the pNUS-GFPcN cloning vector^62^ using NdeI and BglII as restriction sites to generate the gene coding for the CD136-219-GFP fusion protein (36 kDa). The same primers were used with LmjMCA-Flag H147A/C202A^41^ as template to generate the gene coding for CD136-218-GFP H147A/C202A fusion protein (36 kDa). The LmjMCA C-terminal domain from aa 299 to 435 was amplified using the primers 299Fwd_NdeI (5′-GCGCATATGGTGCAGGTGCCGC-3′)/435Rev_BglII (5′-CGCAGATCTTTAGCCAGGCGGGAGT-3′) before insertion into the pNUS-GFPcN cloning vector.

The pGL1078 GFP-ATG8 vector allowing the expression of the autophagosome marker ATG8 fused to GFP and hygromycin resistance was constructed by J Mottram (Institute of Infection, Immunity and Inflammation, University of Glasgow) and kindly provided by G van Zandbergen (Paul Ehrlich Institute, Germany).

### Transfection procedure

Logarithmic *L. major* promastigotes were harvested by centrifugation at 600 × *g* for 10 min, washed once in sterile PBS and resuspended at 3 × 10^7^ cells/ml in 100 *μ*l of Human T Cell Nucleofector solution (Lonza, Basel, Switzerland). Cells were transferred to Amaxa electroporation cuvettes maintained at 4 °C and already containing 10 *μ*g of DNA. Cells were then electroporated with the program U-033 on the Nucleofector machine (Amaxa GmbH, Cologne, Germany). Following electroporation, cells were incubated overnight in their culture medium and transfectants were selected with 30 *μ*g/ml hygromycin B (Life Technologies, France) for single transfection and with 30 *μ*g/ml hygromycin B and 15 *μ*g/ml blasticidin (Life Technologies, France) for double transfections.

### Induction of cell death and autophagy

Cell death was induced by harvesting logarithmic *L. major* cells by centrifugation at 600 × *g* for 10 min and incubating cells at 10^7^ cells/ml in culture medium with 40 *μ*M miltefosine (Santa Cruz Biotechnology, Dallas, TX, USA) for 24 h, 50 *μ*M curcumin (Sigma-Aldrich, St. Louis, MO, USA) or 0.5 mM H_2_O_2_ (Sigma-Aldrich) for 5 h.

For nutrient deprivation, logarithmic *L. major* cells, after harvesting, were washed once with sterile PBS and incubated at 10^7^ cells/ml in a serum-deprived medium for 4 days, possibly with 10 *μ*M wortmannin (Sigma-Aldrich). Cell concentration was evaluated using a Thoma counting chamber.

### Western blot

Twenty micrograms of proteins were separated by SDS-PAGE. Low-range molecular weight standards were used (Bio-Rad Laboratories, Hercules, CA, USA). Proteins were then transferred to a nitrocellulose membrane by electroblotting and incubated with a mouse monoclonal anti-GFP antibody (Roche Diagnostics AG, Basel, Switzerland). Membrane was then incubated with the corresponding secondary antibody coupled to horseradish peroxidase (Promega, Madison, WI, USA) and developed by enhanced chemiluminiscent staining using ECL western blotting system (Amersham Biosciences, Piscataway, NJ, USA).

### Mitochondrial membrane potential

Logarithmic phase promastigotes were incubated with 0.5 mM H_2_O_2_. Cells were collected every hour, cell death induction was stopped with 250 U/ml catalase and cells were then incubated with 500 nM tetramethylrhodamin methyl esther perchlorate (TMRM) for 30 min and analyzed by C6 ACCURI flow cytometer. Fluorescence was detected in FL2. The experiments were done in triplicate, normalized to 100% for untreated parasites.

To test viability, parasites were stained in parallel with the LIVE/DEAD Assay (Invitrogen AG, Switzerland) using a 1 : 1000 dilution. Fluorescence was detected in FL4.

### TUNEL

To detect DNA double-strand breaks, we applied the TUNEL test using the *in situ* cell death detection kit, fluorescein (Roche, Meyla, France). Cells were fixed with paraformaldehyde 4%, laid on an immunoslide and permeabilized with a 0.1% triton and 0.1% sodium citrate solution. The reaction solution from the kit was then added, before observation with a BX51 fluorescence microscope (Olympus, Rungis, France). Bright field and fluorescence images were acquired using the fluorescence imaging system Cell^A^ (Olympus).

### Reverse transcription quantitative PCR

For RNA extraction, the RNeasy Plus mini kit was used (Qiagen, Courtaboeuf, France). Cells were collected by centrifugation at 600 × *g* for 10 min and lysed with the RLT-Plus solution. After passing through a gDNA eliminator column, cells were washed with ethanol 70%, RW1 and RPE buffers. The concentration of the eluated RNAs was evaluated thanks to a NanoVue Plus spectrophotometer (GE Healthcare, Vélizy-Villacoublay, France) before being aliquoted and conserved at −80 °C.

One-step reverse transcription was performed using the high-capacity cDNA reverse transcription kit (Applied Biosystems, Foster City, CA, USA). RNA (10 *μ*l) was added to the same volume of RT-PCR mix containing RT buffer, dNTPs, random primers and the multiscribe reverse transcriptase. Reverse transcription was performed using the following cycling conditions: 10 min at 25 °C, 120 min at 37 °C and 5 min at 85 °C.

For quantitative PCR, the primers 5′-CGAGACTCGGAAGAGAAGTA-3′ and 5′-CTACGAGCATGAGGAAGAGA-3′, targeting the catalytic domain of LmjMCA, were added to the LightCycler 480 Sybr Green I master mix (Roche, Mannheim, Germany). cDNA (5 *μ*l) was added to 20 *μ*l of PCR mix and placed in a Light Cycler 480 with the following cycling conditions: Taq polymerase activation at 95 °C for 10 min and 45 cycles of amplification of 15 s at 95 °C and 60 s at 60 °C. The *kmp11* (Kinetoplastid Membrane Protein 11) gene was used as control, having the same level of expression in WT, apoptotic and autophagic conditions. Ratios of Lmjmca/kmp11 expression were calculated using the Pfaffl method where: ratio=(eff_Lmjmca_)^ΔCqLmjmca^(control-treated)/(eff_kmp11_)^ΔCqkmp11^(control-treated) with ‘eff' the efficiency, ‘control' the WT condition and ‘treated' the apoptotic or autophagic condition. The PCR efficiency of *kmp11* and *Lmjmca* were determined using the serial dilution method on the basis of a linear regression slope.

### Yeast two-hybrid assay

#### Cloning of *L. major* metacaspase gene in pGBKT7 vector for the expression of LmjMCA protein fused to a GAL4 DNA-binding domain (DNA-BD)

The coding sequence of LmjMCA (GeneDB name: *LmjF.35.1580*) in the pESC-His vector (Stratagene, Santa Clara, CA, USA) and mutated in the catalytic dyad (H147A and C202A) was amplified by PCR with primers BD147202MCAfw (5′-CCGCATATGGCAGACCTTTTTGATATTTGG-3′) and BD147202MCArv (5′-CCGGAATTCTTAGCCAGGCGGGAGTGG-3′). The coding sequence of the putative catalytic domain of LmjMCA in the pESC-His vector and mutated in the catalytic cysteine (C202A) was amplified with primers BD202CDfw (5′-CCGCATATGGCGCTTTTCATCGGAATCAA-3′) and BD202CDrv (5′-CCGGAATTCTTATACCTGTTGCATGTACTC-3′). The PCR products were digested with *NdeI* and *EcoRI* restriction enzymes and cloned into the pGBKT7 vector (Clontech, Saint-Germain-en-Laye, France) to generate constructs BD-cMyc-147/202LmjMCA and BD-cMyc-202 cd-LmjMCA, which code for the N-terminally DNA-BD-fused and c-Myc-tagged inactive complete sequence and catalytic domain of LmjMCA, respectively. Both constructs were verified by sequencing (Fasteris SA, Plan-les-Ouates, Switzerland).

In the same manner, the N-terminal region, the region comprising the catalytic domain (mutated in the catalytic dyad) and the C-terminal region of LmjMCA were fused to a GAL4 DNA-binding domain.

#### Construction of GAL4 Activating domain (AD) fusion libraries with ds cDNA from different life cycle stages of *L. major* parasites and two-hybrid screening

GAL AD fusion libraries were produced by co-transforming competent AH109 yeast cells (MATa, trp1-901, leu2-3, 112, ura3-52, his3-200, gal4Δ, gal80Δ, LYS2::GAL1_UAS_-GAL1_TATA_-HIS3, GAL2_UAS_-GAL2_TATA_-ADE2, URA3::MEL1_UAS_-MEL1_TATA_-lacZ, MEL1; Clontech) with CDSIII/SMARTIII ds cDNAs and SmaI-linearized pGADT7-Rec vector. The linear plasmid was restored to its circular form by *in vivo* homologous recombination with overlapping sequences at the ends of the CDSIII/SMARTIII ds cDNAs. Successful plasmid assembly resulted in Leu2^+^ transformants. These cells were also co-transfected either with the BD-cMyc-147/202LmjMCA or BD-cMyc-202 cd-LmjMCA constructs, which resulted in Leu2^+^/Trp1^+^ transformants.

In the same manner, the N-terminal region, the region comprising the catalytic domain (mutated in the catalytic dyad) and the C-terminal region of LmjMCA were fused to a GAL4-activating domain and competent AH109 yeast cells were co-transfected with the AD-LmjMCA and BD-LmjMCA constructs to study self-interaction of LmjMCA.

Positive interactions between GAL4-AD and DNA-BD fused proteins resulted in the transcription activation of the reporter genes: *HIS3, ADE2, lacZ and MEL1* (Leu2^+^/Trp1^+^/His3^+^/Ade2^+^/Galactosidase^+^ transformants). Transfected yeast cells were initially cultured on synthetic/dropout plates without leucine, tryptophan and histidine (SD/DO/-Leu/-Trp/-His plates) consisting of 0.67% yeast nitrogen base, 2% glucose as carbon source, 2% agar and an amino acid solution with 20 mg/l adenine, arginine, methionine and uracil; 30 mg/l isoleucine, lysine and tyrosine; 50 mg/l phenylalanine; 150 mg/l valine; and 200 mg/l threonine. Plates were incubated at 30 °C for 4 days. Obtained colonies were replicated afterwards on SD/DO/Agar plates without leucine, tryptophan, histidine and adenine, and with X-α-Gal (SD/DO/-Leu/-Trp/-His/-Ade/X-α-Gal plates), where true positive interactions induced the formation of large blue colonies and incubated at 30 °C for 4 days.

#### Identification of proteins interacting with LmjMCA

Double-stranded cDNAs inserted into the pGADT7-Rec vector were amplified from Leu2^+^/Trp1^+^/His3^+^/Ade2^+^/Galactosidase^+^ colonies by PCR. For that, we tested 25% of colonies obtained with 147/202LmjMCA and logarithmic and stationary promastigotes and axenic amastigotes and all colonies obtained with 202 cd-LmjMCA and with 147/202LmjMCA and the intracellular amastigotes library. Yeast cells were scraped from a colony and diluted in a PCR master mix containing the primers 5′-LD amplimer (5′-CTATTCGATGATGAAGATACCCCACCAAACCC-′3) and 3′-LD amplimer (5′-GTGAACTTGCGGGGTTTTTCAGTATCTACGAT-′3). PCR products were separated by electrophoresis in 0.8% agarose gels with 0.5 *μ*g/ml ethidium bromide, purified with a Wizard SV Gel and PCR Clean-up System (Promega), and sent for sequencing with a standard T7 primer (5′-TAATACGACTCACTATAGG-′3; Fasteris). Obtained gene sequences were translated to protein conserving the reading frame after the AD GAL4-activating domain (http://www.expasy.org/tools/dna.html) and analyzed by BLASTP (http://www.genedb.org/genedb/leish/blast.jsp) against a database of *L. major* predicted proteins (www.genedb.org).

#### Confirmation by plasmid isolation of proteins interacting with LmjMCA

Overnight cultures of yeast colonies where a *L. major* protein was identified as interacting with LmjMCA, were used for plasmid isolation. Yeast cells were diluted in a solution containing 1 M sorbitol and 100 mM EDTA, pH 8.0 and treated with 0.5 *μ*g/*μ*l Zymolyase 20 T (Seikayaku) at 37 °C for 1 h. Yeast spheroplasts were diluted in a solution containing 50 mM TrisCl and 20 mM EDTA pH 8.0 and lysed with 1% SDS at 65 °C for 30 min. Yeast DNA was precipitated with 1 M potassium acetate and 50% isopropanol. Obtained DNA was then used to transform Top10 *E. coli* cells, which were cultured in the presence of ampicillin to select and purify the pGADT7-Rec constructs with a Wizard Plus SV Minipreps DNA purification System (Promega). Gene inserts were sequenced with a standard T7 primer (forward) and a 3′-AD primer (reverse) (5′-AGATGGTGCACGATGCACA-′3). Obtained sequences were translated to protein conserving the reading frame after the AD GAL4-activating domain and analyzed by BLASTP against a database of *L. major* predicted proteins. These results were then compared with the previously obtained by PCR amplification.

### Co-immunoprecipitation

Yeast cells from the yeast two-hybrid assay were lysed with 1% Triton X-100 containing buffer and glass beads. The lysed cells were then incubated with protein-Sepharose beads coupled to a myc-antibody. Lysates (20 *μ*g) and the immunoprecipitated beads were electrophorized on a 10% SDS-PAGE gel. Proteins were transferred to a nitrocellulose membrane by electroblotting and probed with an HA antibody. Blots were exposed to the corresponding horseradish peroxidase-conjugated secondary antibody (Promega) and developed by enhanced chemiluminiscent staining using ECL western blotting system (Amersham Biosciences). Low-range molecular weight standards were used as reference proteins (Bio-Rad Laboratories).

### Pull-down assay

Parasites transfected with GFP-tagged *L. major* MPK7 (GFPK7) were grown in the presence of 100 mg/ml of geneticin,^63^ and the fusion protein and its interaction partners were isolated from crude cell lysates with a *μ*MACS GFP Isolation Kit (Miltenyi Biotec, Auburn, CA, USA). Briefly, 10^9^ WT or GFPK7 cells were washed once by centrifugation with ice-cold PBS containing a cocktail of protease inhibitors (Complete Mini tablets, Roche Applied Science, Indianapolis, IN, USA), lysed in 1 ml lysis buffer and subjected to magnetic separation according to the manufacturer's specifications. Equal amounts of total proteins were incubated with 50 *μ*l of magnetic bead-conjugated mouse monoclonal anti-GFP antibody for 1 h at 4 °C, and immunocomplexes were immobilized on the mMACS separator, washed and eluted with 2 × 50 *μ*l heated elution buffer provided by the kit. Fifteen microliters of immunocomplexes were separated by SDS-PAGE on NuPAGE 4–12% Bis-Tris gels (Invitrogen, France) and blotted onto polyvinylidene difluoride (PVDF) membranes (Pierce Protein, Rockford, IL, USA). Proteins were revealed using anti-GFP (Miltenyi Biotec) and anti-MCA antibodies (J. Mottram, Institute of Infection, Immunity and Inflammation, University of Glasgow).

### Statistical analysis

For statistics, unpaired *t*-tests were realized. Results were considered statistically significant when *P*<0.05. For significant differences, *P<0.05, **P<0.01 and ***P<0.001.

## Figures and Tables

**Table 1:** Hypothetical proteins of *L. major* parasites identified as interacting with LmjMCA

	**Protein libraries**	**Reference of hypothetical protein sequence and predicted functional domain**^a^	
147/202LmjMCA	Logarithmic promastigotes	LmjF.24.1570: RNA-binding domain	LmjF.21.0820 LmjF.26.2650
	Stationary promastigotes	LmjF.10.0620: twin arginine translocation signal domain LmjF.17.1270: ferredoxin domain LmjF.19.0330: WD40 repeat LmjF.31.1320: phosphatase domain LmjF.35.1620: concanavalin A/glucanase domain LmjF.35.4350: signal peptide/PGAP1-like domain LmjF.36.3340: signal peptide LmjF.25.2200: Parkin co-regulated protein domain **LmjF.35.0610: signal peptide**	LmjF.21.0905 **LmjF.22.0100** LmjF.23.1075 LmjF.27.0850 LmjF.30.3620 **LmjF.31.1540** LmjF.30.1390 LmjF.36.5210
	Axenic amastigotes	LmjF.06.0850: signal peptide LmjF.18.1050: heat shock protein DnaJ domain LmjF.19.1410: polyA polymerase domain **LmjF.25.0770: signal peptide (4)** LmjF.27.0020: signal peptide/EGF-like domain LmjF.30.0950: WD40 repeat LmjF.30.1190: transcription factor domain LmjF.30.1810: signal peptide/PapD-like domain LmjF.30.2390: signal peptide/RNI-like domain LmjF.30.3310: MORN motif (2) LmjF.31.0680: C2 domain LmjF.31.1020: signal peptide/GPI-anchor LmjF.31.1210: ubiquitin domain/zinc finger, RanBP2-type LmjF.31.1330: nucleotide binding LmjF.33.1460: RNA binding LmjF.33.1490: tetratricopeptide repeat LmjF.35.1020: Zn finger (3) LmjF.35.1040: Zn finger	LmjF.14.0300 LmjF.19.0020 (2) LmjF.20.1700 (2) **LmjF.22.0100** **LmjF.31.1540** LmjF.34.1370 LmjF.09.1300 LmjF.16.1210 LmjF.25.1350 LmjF.31.0210 LmjF.35.0960
	Intracellular amastigotes	LmjF.22.0410: signal peptide/mannose-6-phosphate receptor binding domain/growth factor receptor domain LmjF.24.1390: Zn finger/EF hand domain **LmjF.25.0770: signal peptide** **LmjF.35.0610: signal peptide**	**LmjF.22.0100** LmjF.22.1440
202 cd-LmjMCA	Stationary promastigotes	LmjF.15.0310	

Reiterative proteins were found in more than one life cycle stage of *L. major* parasites (in bold), and in two (2), three (3) or four (4) yeast colonies

a tritrypdb.org

**Table 2:** Proteins of *L. major* parasites with function inferred from homology, experimental characterization or previous publication, identified as interacting with LmjMCA

	**Protein library**	**Identified proteins**^a^	**Total length in amino acids**	**Amino acids interacting with LmjMCA**
147/202LmjMCA	Logarithmic promastigotes	**LmjF.13.1640: mitogen-activated protein kinase 7, putative**	605	251–350: catalytic
		LmjF.18.0510: aconitase, putative	896	470–650: signature
		LmjF.36.3910: S-adenosyl homocysteine hydrolase	437	100–370: signature
	Stationary promastigotes	**LmjF.13.1640: mitogen-activated protein kinase 7, putative**	605	251–350: catalytic
		LmjF.26.1620: Cdp-diacylglycerol synthetase-like protein	431	290–431: signature
		**LmjF.27.0500: calpain-like cysteine peptidase, putative**	6164	135–250: catalytic
		LmjF.36.3150: ADP-ribosylation factor GTPase activating protein, putative	418	380–410: C-terminus
		LmjF.36.6430: transport protein sec23-like	850	20–180: Zn finger
		LmjF.36.6460: tartrate-sensitive acid phosphatase acp-3.2, putative (2)	224	30–190: signature
	Axenic amastigotes	LmjF.04.0330: mitochondrial exoribonuclease DSS-1, putative	857	450–600: catalytic
		LmjF.06.0950: glucosamine-fructose-6-phosphate aminotransferase, putative	670	90–310: signature
		LmjF.09.0360: DNA photolyase, putative	934	660–934: signature
		LmjF.23.0730: RNA-binding protein, putative	599	420–599: C-terminus
		LmjF.26.2440: protein kinase, putative (2)	1043	600–860: signature
		**LmjF.27.0500: calpain-like cysteine peptidase, putative**	6164	847–1040: catalytic
		LmjF.32.1400: DEAD/DEAH box helicase-like protein, putative	1691	1380–1650: C-terminus
		LmjF.36.0150: fructose-6-phosphate2-kinase/fructose-2,6-bisphosphatase-like protein	485	210–410: signature
		LmjF.36.5560: aminopeptidase P1, putative	840	660–830: C-terminus
	Intracellular amastigotes	LmjF.05.0530: kinetoplast-associated protein-like protein	2061	1910–2061: C-terminus
		LmjF.22.1110: dynein heavy chain, cytosolic, putative	5635	4350–4550: signature
		LmjF.32.2950: nucleoside diphosphate kinase b	151	10–151: signature
202 cd-LmjMCA	Axenic amastigotes	LmjF.10.1160: Rab1 small GTP-binding protein	216	70–100: signature

Reiterative proteins were found in more than one life cycle stage of *L. major* parasites (in bold), and in two (2) independent colonies

a tritrypdb.org

**Table 3:** Classification and intracellular localization of *L. major* proteins interacting with LmjMCA^a^

**Group**	**Protein**	**Intracellular localization**
Ser/Thr protein kinases	LmjF.13.1640: mitogen-activated protein kinase 7, putative	Cytoplasm
	LmjF.26.2440: protein kinase, putative	Cytoplasm
Proteases	**LmjF.27.0500: calpain-like cysteine peptidase, putative**	Cytoplasm
	LmjF.36.5560: aminopeptidase P1, putative	GPI-anchored
Vesicle transport	LmjF.10.1160: Rab1 small GTP-binding protein	Cytoplasm
	LmjF.22.1110: dynein heavy chain, cytosolic, putative	Cytoplasm
	LmjF.36.3150: ADP-ribosylation factor GTPase activating protein, putative	Cytoplasm
	LmjF.36.6430: transport protein sec23-like	Cytoplasm
Metabolism	LmjF.06.0950: glucosamine-fructose-6-phosphate aminotransferase, putative	Cytoplasm
	LmjF.18.0510: aconitase, putative	Cytoplasm/mitochondrion
	LmjF.26.1620: Cdp-diacylglycerol synthetase-like protein	Membrane
	LmjF.32.2950: nucleoside diphosphate kinase b	Cytoplasm
	LmjF.36.0150: fructose-6-phosphate2-kinase/fructose-2,6-bisphosphatase-like protein	Cytoplasm
	LmjF.36.3910: S-adenosyl homocysteine hydrolase	Cytoplasm
	LmjF.36.6460: tartrate-sensitive acid phosphatase acp-3.2, putative	Cytoplasm
Nucleotide binding	LmjF.04.0330: mitochondrial exoribonuclease DSS-1, putative	Mitochondrion
	LmjF.05.0530: kinetoplast-associated protein-like protein	Kinetoplast
	LmjF.09.0360: DNA photolyase, putative	Nucleus
	LmjF.23.0730: RNA-binding protein, putative	GPI-anchored
	LmjF.32.1400: DEAD/DEAH box helicase-like protein, putative	Cytoplasm/nucleus

Reiterative proteins were found in more than one life cycle stage of *L. major* parasites (in bold)

a tritrypdb.org

## Abbreviations

AD: activating domain
Arf: ADP-ribosylation factor
BD: binding domain
CALP: calpain-like cysteine peptidase
CD: catalytic domain
COPII: coat protein complex II
FCS: fetal calf serum
GAP: GTPase activating protein
MCA: metacaspase
MLS: mitochondrion localization signal
PCD: programmed cell death
RT-qPCR: reverse transcription quantitative PCR
WT: wild-type
