# Supplementary figures and images for: Implication of different domains of the Leishmania major metacaspase in cell death and autophagy
Source: Cell Death Dis. 2015 Oct 22;6(10):e1933–. doi: 10.1038/cddis.2015.288 (PMC4632311; doi:10.1038/cddis.2015.288)

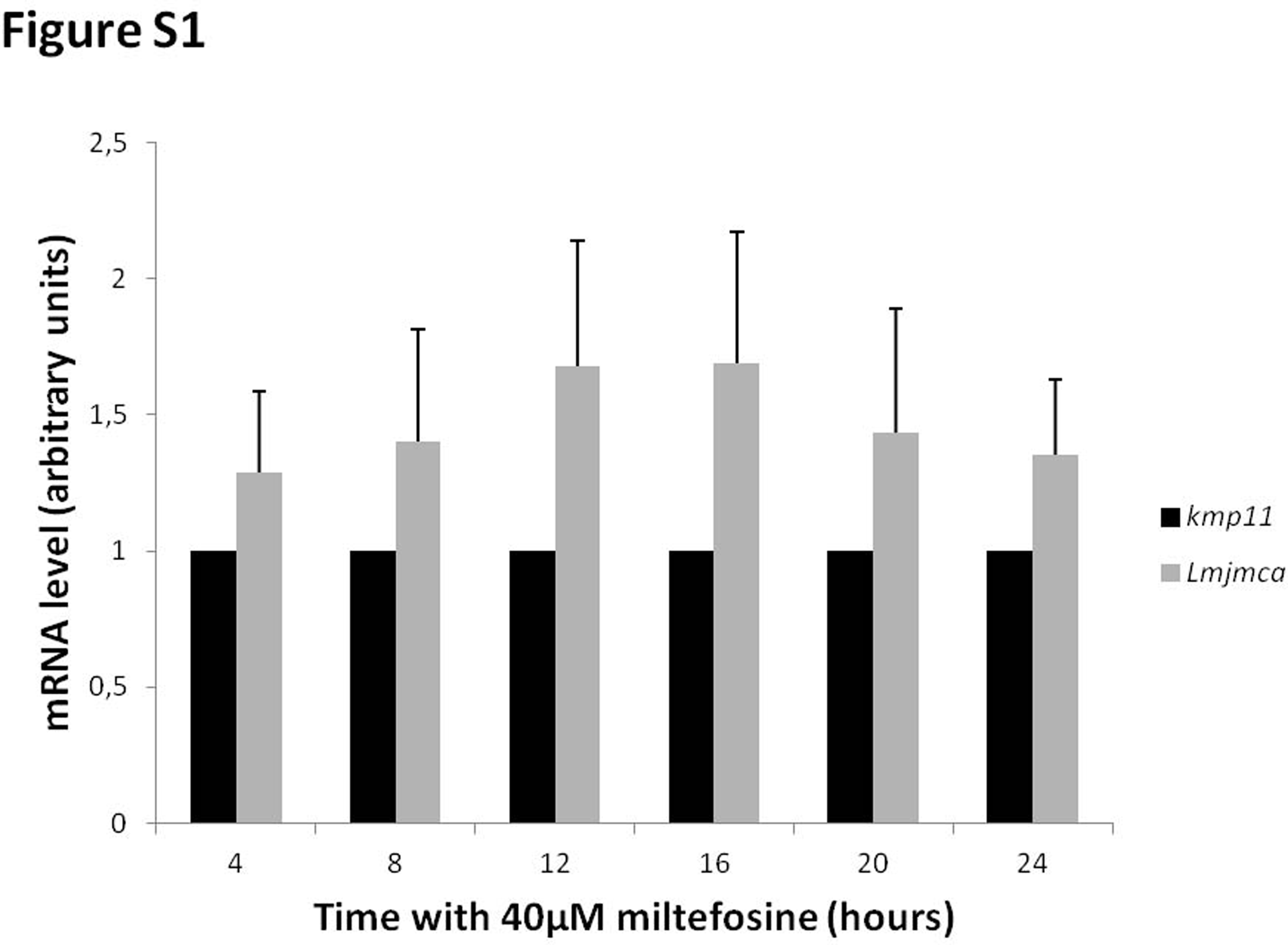

Supplement: Supplementary Figure S1 [file cddis2015288x1.tif]

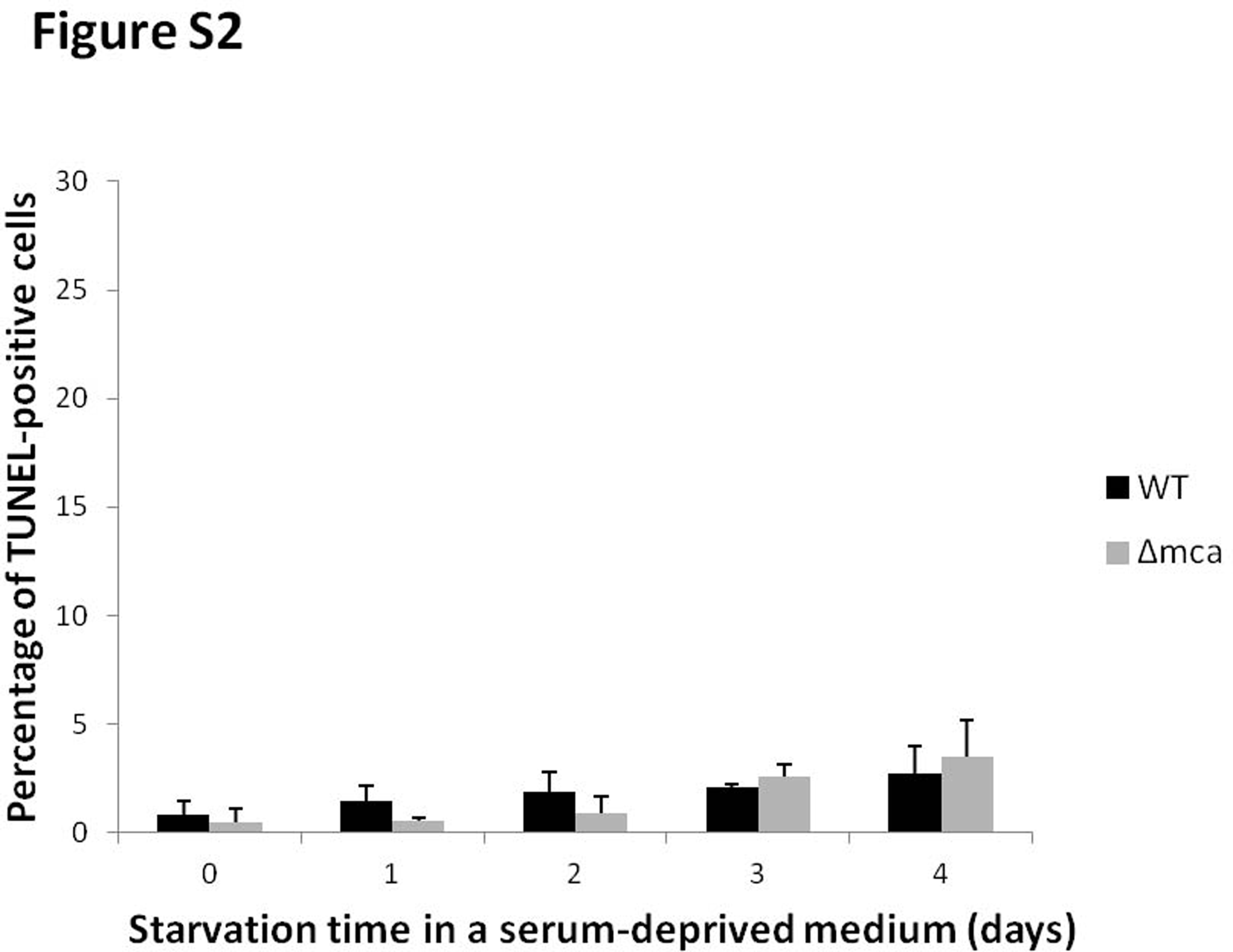

Supplement: Supplementary Figure S2 [file cddis2015288x2.tif]
