# Supplementary material for: Implication of different domains of the Leishmania major metacaspase in cell death and autophagy
Source: Cell Death Dis. 2015 Oct 22;6(10):e1933–. doi: 10.1038/cddis.2015.288 (PMC4632311; doi:10.1038/cddis.2015.288)
Supplement: Supplementary Figure Legends [file cddis2015288x3.doc]

**Figure S1. LmjMCA involvement in cell death is not linked to gene overexpression.**

RT-qPCR quantification of *kmp11* (Kinetoplastid Membrane Protein, used as a control) and *Lmjmca* mRNA expression, after culture of WT cells with 40µM of miltefosine (means ± sd from five independent experiences). No significant *Lmjmca* overexpression could be detected.

**Figure S2. Cultivation of cells in a serum-deprived medium induced no cell death.**

Percentage of TUNEL-positive WT (dark) and LmjMCA-deficient (grey; Δ*mca*) cells cultivated in a serum deprived medium: means ± sd from three independent experiences. No TUNEL-positive cells appeared when WT and Δ*mca* cells were cultivated in a serum-deprived medium.
