# Supplementary material for: Implication of different domains of the Leishmania major metacaspase in cell death and autophagy
Source: Cell Death Dis. 2015 Oct 22;6(10):e1933–. doi: 10.1038/cddis.2015.288 (PMC4632311; doi:10.1038/cddis.2015.288)
Supplement: Supplementary Table S1 [file cddis2015288x4.doc]

**Supplementary Table S1. *L. major* polypeptides with functions inferred from homology and interacting with full length 147/202 LmjMCA or catalytic 202cd-LmjMCA**

| **Bait** | **Library** | **Identified proteins *** | **Length (in AA)** | **Interacting region**  **Start-End**** | **%***** | **E-value** | **Id.** | **Notes** |
| --- | --- | --- | --- | --- | --- | --- | --- | --- |
| 147/202 LmjMCA | Logarithmic  promastigotes | LmjF.13.1640: Mitogen-activated protein kinase 7 | 605 | 251-350 | 16.3 | 4e-56 | 99 | (1)(2) |
|  |  | LmjF.18.0510: aconitase | 896 | 470-650 | 20.0 | 2e-115 | 100 |  |
|  |  | LmjF.36.3910: S-adenosyl homocysteine hydrolase | 437 | 100-370 | 61.8 | 1e-180 | 99 |  |
|  | Stationary  promastigotes | LmjF.13.1640: Mitogen-activated protein kinase 7 | 605 | 251-350 | 16.3 | 2e-57 | 100 | (1) |
|  |  | LmjF.26.1620: Cdp-diacylglycerol synthetase-like protein | 431 | 290-431 | 32.7 | 2e-97 | 99 |  |
|  |  | LmjF.27.0500: calpain-like cysteine peptidase | 6164 | 135-250 | 1.8 | 1e-71 | 100 | (1)(3)(4) |
|  |  | LmjF.36.3150: ADP-ribosylation factor GTPase activating protein | 418 | 380-410 | 7.17 | 3e-81 | 99 |  |
|  |  | LmjF.36.6430: transport protein sec23-like | 850 | 20-180 | 14.1 | 2e-106 | 100 |  |
|  |  | LmjF.36.6460: tartrate-sensitive acid phosphatase acp-3.2 | 224 | 30-190 | 71.4 | 1e-102 | 98 | (4) |
|  | Axenic amastigotes | LmjF.04.0330: mitochondrial exoribonuclease DSS-1, putative | 857 | 450-600 | 17.5 | 1e-94 | 99 |  |
|  |  | LmjF.06.0950: glucosamine-fructose-6-phosphate aminotransferase | 670 | 90-130 | 5.97 | 8e-153 | 99 |  |
|  |  | LmjF.09.0360: DNA photolyase | 934 | 660-934 | 29.3 | 7e-170 | 98 |  |
|  |  | LmjF.23.0730: RNA-binding protein | 599 | 420-599 | 29.8 | 2e-114 | 99 |  |
|  |  | LmjF.26.2440: protein kinase | 1043 | 600-860 | 24.9 | 3e-147 | 99 | (4) |
|  |  | LmjF.27.0500: calpain-like cysteine peptidase | 6164 | 847-1040 | 3.1 | 5e-131 | 99 | (1)(3) |
|  |  | LmjF.32.1400: DEAD/DEAH box helicase-like protein | 1691 | 1380-1650 | 15.9 | 1e-160 | 99 |  |
|  |  | LmjF.36.0150 : fructose-6-phosphate2-kinase/ fructose-2,6-bisphosphatase-like-protein | 485 | 210-410 | 41.2 | 2e-139 | 99 |  |
|  |  | LmjF.36.5560: aminopeptidase P1 | 840 | 660-830 | 20.2 | 1e-121 | 100 |  |
|  | Intracellular  Amastigotes | LmjF.05.0530: kinetoplast-associated protein-like protein | 2061 | 1910-2061 | 7.32 | 4e-94 | 100 |  |
|  |  | LmjF.22.1110: dynein heavy chain, cytosolic | 5635 | 4350-4550 | 3.5 | 2e-132 | 99 |  |
|  |  | LmjF.32.2950: nucleoside diphosphate kinase b | 151 | 10-151 | 93.4 | 1e-93 | 99 |  |
| 202cd-LmjMCA | Axenic amastigotes | LmjF.10.1160: Rab1 small GTP-binding protein | 216 | 70-100 | 13.9 | 3e-11 | 96 |  |

*tritrypdb.org; **: position of the amino acid in the full length amino acid sequence; %: percentage of overlapping sequences between the identified polypeptide and the full length sequences of *L. major* polypeptides retrieved from the *L. major* *Friedlin* genome database; ******* : no gaps were found in the overlapping sequences; E-value: based on blastp of *L. major* *Friedlin* genome database; Id.: percentage of identity between the interacting *L. major* amino acid sequences and *L. major* amino acid sequences retrieved from the *L. major Friedlin* genome database; (1) Reiterative proteins were found in more than one life cycle stage of *L. major* parasites; (2) confirmed by immuno-precipitation (IP) and pull-down assay; (3) confirmed by IP; (4) interacting partner identified in two independent colonies.
